# Spectrum of sensitive skin in India: a collaborative expert position statement

**DOI:** 10.3389/fmed.2025.1625172

**Published:** 2025-08-26

**Authors:** Deepika Pandhi, Arun C. Inamadar, Abir Saraswat, Abhishek De, Anchala Parthasaradhi, B. S. Chandrasekhar, Malavika Kohli, Manjunath Shenoy, Murlidhar Rajagopalan, Pravin Banodkar, Saumya Panda, Shital Poojary, Priti Thakor, Ruchi Shah, Someshwar Rayasam

**Affiliations:** ^1^Department of Dermatology, University College of Medical Sciences & GTBH, Delhi, India; ^2^Sri B. M. Patil Medical College, Hospital & Research Center, BLDE University, Vijaypur, India; ^3^Midland Hospital & Research Centre, Lucknow, India; ^4^CNMC, Kolkata, India; ^5^Anchalas Skin Institute, Hyderabad, India; ^6^CUTIS Academy of Cutaneous Sciences, Bengaluru, India; ^7^Skin Secrets, Mumbai, India; ^8^Yenepoya Medical College, Mangalore, India; ^9^Apollo Children’s Hospital and Fetal Care Research Center, Chennai, India; ^10^Skin Crest Clinic, Mumbai, India; ^11^Belle Vue Clinic, Kolkata, India; ^12^K. J. Somaiya Medical College, Mumbai, India; ^13^JNTL Consumer Health (India) Private Limited, Mumbai, India

**Keywords:** oatmeal-based moisturizers, sensitive skin spectrum, emollients, cosmeceuticals, skin barrier, filaggrin gene, trans epidermal water loss

## Abstract

**Introduction:**

Sensitive skin poses a significant dermatological concern in India, influenced by diverse climatic conditions, cultural practices, and pollution levels. These factors contribute to the prevalence of sensitive skin, which is characterized by symptoms such as redness, itching, and dryness. Sensitive skin is better understood as a distinct syndrome rather than a linear progression from dry skin to conditions like acne, atopic dermatitis, and rosacea. The condition impacts the overall quality of life of affected individuals, making it crucial to understand its spectrum and management.

**Methods:**

A panel of 12 dermatologists from various regions of India provided expert insights into the characterization of sensitive skin. The dermatologists discussed the spectrum of sensitive skins and provided strategies for managing the condition. Their recommendations emphasized the importance of individualized skincare regimens tailored to the specific needs of patients.

**Results:**

Several studies have discussed the heterogeneity of sensitive skin, indicating the complexity of its diagnosis and management. The market is saturated with a variety of cosmeceuticals aimed at addressing sensitive skin. However, there are no established guidelines for using these products, leading to the adoption of individualized skincare regimens as the most common approach.

**Discussion:**

This expert paper highlights the necessity of recommending cosmeceuticals in managing sensitive skin and emphasizes the importance of personalized skincare regimens. By comprehensively addressing the spectrum of sensitive skin, clinicians can prevent the progression to severe dermatological conditions, thereby safeguarding the skin barrier and improving the overall quality of life for individuals affected by sensitive skin.

## 1 Introduction

Sensitive skin is a dermatological concern that transcends geographical boundaries, impacting individuals across diverse populations. In India, a country known for its rich cultural heritage and climatic diversity, the issue of sensitive skin presents unique challenges due to complex interactions between climate, lifestyle, and traditional skincare practices (1). It is a relatively common condition with studies reporting a prevalence of 30–40% in the Indian population (2). A nationally representative survey of over 3,000 adults found that 27.9% of men and 36.7% of women reported having ‘sensitive’ or ‘very sensitive’ skin, highlighting its widespread impact. The high prevalence, combined with heightened reactivity to environmental factors, skincare products, and stressors, underscores the need for tailored clinical approaches (1). Understanding the prevalence and nuances of sensitive skin in the Indian context is essential for healthcare providers and individuals seeking effective skincare solutions tailored to the country’s dynamic realities. The rich ethnic and genetic diversity present in India results from millennia of cultural and geographical influences, which have led to differences in various traits, including skin sensitivity (1). Extreme cold or hot temperatures can strip off the natural moisture from the skin, resulting in dry, itchy, and flaky skin. Hot and humid climates can increase the production of sweat and potentially trigger acne or irritation (3). Sensitive skin can be categorized into three different variants based on its epidermal barrier function: Type I refers to low or diminished epidermal barrier function, Type II represents regular barrier function displaying signs of inflammation, and Type III involves normal barrier function with reactivity issues but without inflammation (4).

Sensitive skin syndrome is a condition characterized by the occurrence of unpleasant sensations, such as burning, pain, pruritis, stinging, and tingling sensations, in response to stimuli that normally should not provoke such sensations. Sensitive skin usually presents subjective and objective symptoms. Individuals exhibiting sensitive skin often report sensations such as stinging, tingling, and mild prickling upon the application of specific skincare products or when exposed to environmental elements. These sensations are frequently accompanied by a burning perception, which can intensify if the skin interacts with irritants or aggressive agents. Moreover, pruritus or itchiness represents a vexing symptom frequently identified among those with sensitive skin. The objective perceptions of sensitive skin are assessed through evaluations by medical professionals, and they encompass a comprehensive spectrum of cutaneous reactions (5).

International guidelines reflect a shared global understanding of sensitive skin management. The IFSI recommends avoidance of triggers, application of well-tolerated emollients, and consideration of psychosocial factors in treatment (6). Similarly, the American Academy of Dermatology and the European Academy of Dermatology and Venereology suggest using fragrance-free, low-preservative products, physical sunscreens, gentle cleansing, and prior patch testing for sensitive skin (7, 8). These recommendations support the India-specific framework proposed in this manuscript, highlighting the need for localized adaptation while maintaining international best practices. In this article, we have undertaken a comprehensive examination of the complexities related to the management of sensitive skin issues in the Indian context. Our objectives include distinguishing and clinically diagnosing sensitive skin as opposed to reactive skin and formulating management strategies for sensitive skin across diverse patient profiles. Furthermore, we have assessed the efficacy of colloidal oatmeal-based moisturizers in addressing sensitive skin concerns through insights garnered from expert panel discussions. This article provides an extensive overview of expert opinions and offers recommendations to tackle the challenges associated with managing sensitive skin in India.

## 2 Methodology

An advisory board meeting was convened to gather clinical insights from experts regarding the spectrum of sensitive skin. The advisory board panel comprised 12 doctors specializing in dermatology and related fields of skin sciences. They were carefully selected to ensure a comprehensive representation of knowledge and clinical proficiency in dermatology across India. The formation of the expert committee was devoid of any selection bias. The primary objective of the expert committee meeting was to facilitate an exhaustive discussion and generate expert recommendations concerning the management of sensitive skin in the Indian context. The experts examined scientific evidence and guideline recommendations and integrated their clinical experience into the following key areas:

1. Defining the spectrum of sensitive skin
2. Gaining a comprehensive understanding of the spectrum of sensitive skin within the Indian population
3. Discerning the clinical characteristics and diagnostic criteria to differentiate sensitive skin from reactive skin
4. Formulating management strategies for sensitive skin in diverse patient profiles
5. Reviewing clinical evidence on the role of colloidal oatmeal in the management of sensitive skin.

Prior to the group discussion, there was validation of questions and responses stratification. Following the group discussion, expert opinions and recommendations were formulated based on the collective opinions and agreements of the majority of people. To support these discussions, a comprehensive literature review was conducted using data obtained from reputable sources, including the PubMed Database, Google Scholar, and the Cochrane Library, to identify relevant articles published between June 1997 and June 2023. The search strategy employed relevant free-text keywords combined with appropriate Boolean operators (AND, OR). Keywords utilized in the search included terms such as “Indian skin,” “sensitive skin,” “cosmeceuticals,” “oatmeal-based moisturizers,” “emollients,” “filaggrin gene,” “skin barrier,” “guidelines,” and “management.” The expert opinion paper incorporated content from a diverse range of sources, including randomized controlled trials, case reports, practice guidelines, systematic literature reviews, and meta-analyses.

## 3 Results and discussion

### 3.1 Spectrum of sensitive skin

**Figure 1:**
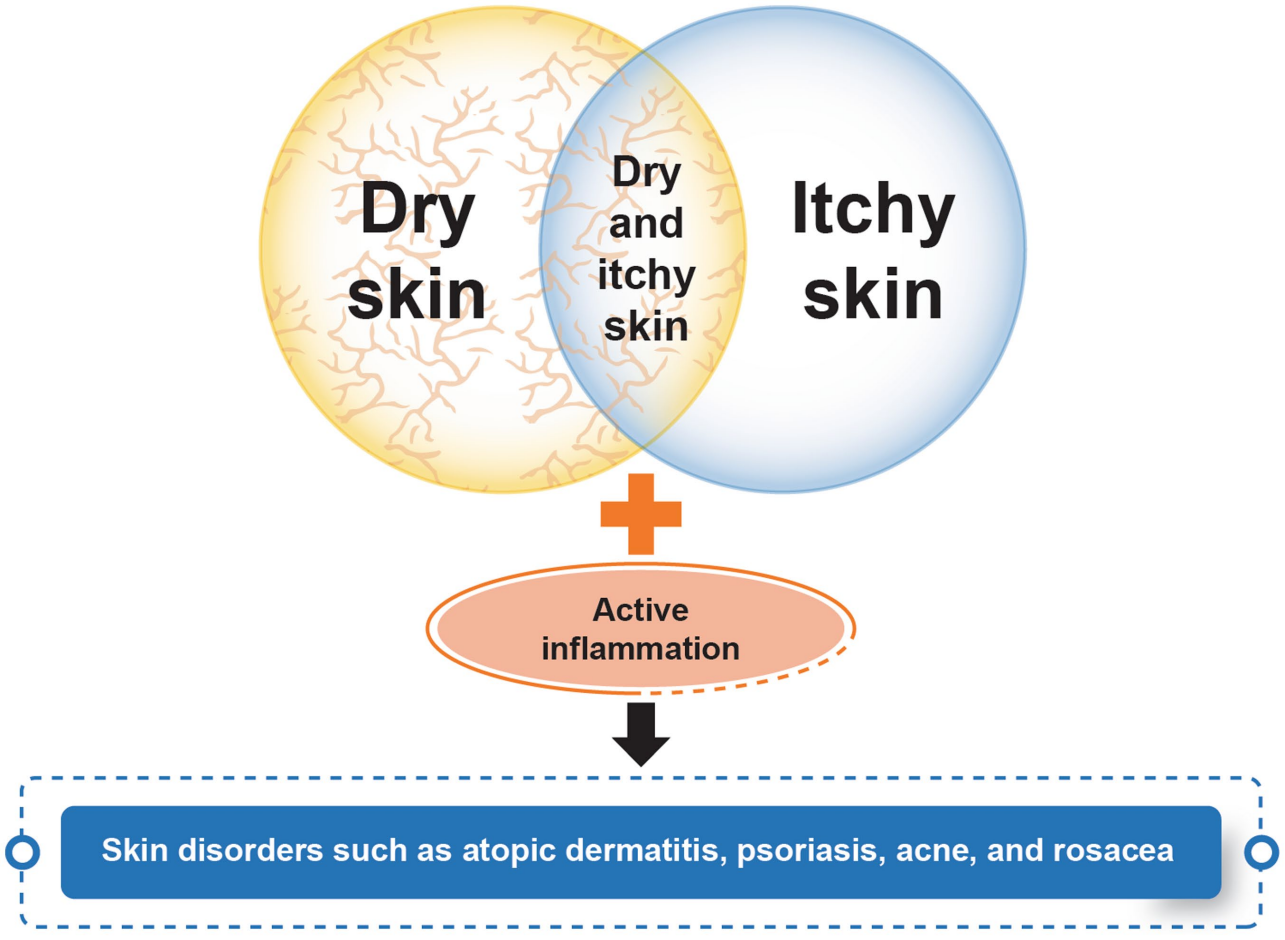
The spectrum of sensitive skin.

Sensitive skin is a multifaceted phenomenon that defies a linear progression spectrum from mild dryness to more severe conditions, such as atopic dermatitis, psoriasis, acne, and rosacea. It can be better understood as a syndrome encompassing a range of interconnected factors as presented in Figure 1.

#### 3.1.1 Expert recommendation

The expert panel recommends redefining the conceptual model of sensitive skin. It should not be viewed as a linear progression spectrum that starts from dry skin and progresses toward pathological conditions such as atopic dermatitis, psoriasis, acne, or rosacea. Instead, sensitive skin should be understood as a distinct syndrome. This syndrome is best visualized using a Venn diagram, which illustrates the intersection of various factors, including the affected body site and individual responses to the administered questionnaire. This model allows for a more comprehensive and accurate representation of the multifaceted nature of sensitive skin.

### 3.2 Classification of sensitive skin

The sensitive skin phenomenon has been examined and classified by researchers Pons-Guiraud and Muizzuddin, each offering distinct perspectives. Pons-Guiraud’s classification identifies distinct categories within the realm of sensitive skin: Extra sensitive skin’ represents an extreme level of sensitivity; sensitivity to environmental factors’ highlights the skin’s responsiveness to external elements, such as temperature, wind, and pollution; and ‘sensitivity to cosmetics’ signifies a heightened susceptibility to skincare and cosmetic products (9). On the other hand, Muizzuddin’s classification focuses on various specific aspects of sensitive skin: Delicate skin’ emphasizes the fragility of the skin and its propensity to react sensitively to various factors, and ‘reactive skin’ encompasses skin that reacts intensely to stimuli, leading to redness, itching, or inflammation. The term ‘stingers’ denotes those who experience immediate stinging sensations upon contact with certain substances, reflecting an instant and pronounced skin response (10). Based on the presence or absence of associated skin disease, sensitive skin syndrome is classified into primary and secondary sensitive skin syndrome. The primary sensitive skin syndrome is genetically linked, marked by thin, fair skin exhibiting mild telangiectasia and an inherent intolerance to various products. A person with primary sensitive skin syndrome does not have any underlying disease, whereas, in the case of secondary sensitive skin syndrome, the individual may have an underlying skin disease, such as atopic dermatitis, acne, or rosacea (4). In addition to these classifications, sensitive skin has also been categorized by different researchers based on various criteria, such as genetic predisposition, age-related sensitivity, and sensitivity linked to medical conditions, such as rosacea or eczema (4, 9).

### 3.3 Pre-disposing factors contributing to sensitive skin

Sensitive skin is an interplay of 4 pathological factors: Diminished skin barrier function, altered skin immunity, enhanced neurohormonal response, and psychological associations. Recent evidence highlights the role of psychological stress as a contributing factor for sensitive skin. For instance, Manav *et al*. demonstrated a significant association between increased anxiety levels and altered biophysical properties, including heightened skin sensitivity and reactivity. These findings highlight the need for clinicians to consider psychological factors alongside environmental and genetic determinants when evaluating and managing sensitive skin (11).

Table 1 elucidates different factors affecting sensitive skin (5).

**Table 1:** Factors affecting sensitive skin.

Host factors	Impact on sensitive skin
Age	Younger age groups are more prone to skin sensitivity than the elderly.
Gender	Women are affected by skin sensitivity more than men due to hormonal changes (e.g., menstrual cycle) and variation in the epidermal thicknesses (epidermal thickness is greater in males).
Ethnicity	Asians are more prone to skin sensitivity than Caucasians.
Environmental factors	Skin sensitivity is higher in winter than in summer due to reduced hydration in the stratum corneum and increased exposure to sodium lauryl sulfate in winter.
Anatomical site	The heightened susceptibility to skin sensitivity in the facial region can be attributed to the presence of a thin epidermal barrier and a high density of nerve endings.

### 3.4 Structural contributors to sensitive skin

The structural contributors of sensitive skin are rooted in various elements of the skin’s composition and function. A thin stratum corneum (SC), coupled with reduced water content and increased activity of sweat glands can cause skin sensitivity. This combination compromises the skin’s natural barrier, allowing irritants to penetrate more easily and trigger reactions (5, 12). The intricate network of nerve connections within the epidermal layer further contributes to the skin’s sensitivity. An elevation in epidermal innervation, or the presence of more nerve fibers in this layer, can lead to heightened skin sensitivity. This increased innervation can make the skin more responsive to stimuli, amplifying the potential for discomfort and reactions (5). Trans-epidermal water loss (TEWL) plays a significant role in determining the skin’s vulnerability. When TEWL is elevated, indicating an increased loss of water from the skin’s surface, the protective barrier function is compromised. A weakened barrier makes the skin more susceptible to irritants, allergens, and environmental factors, intensifying the likelihood of sensitive reactions (5, 12).

### 3.5 Sensitive skin on the face vs. sensitivity in other body parts

Permission must be obtained for use of copyrighted material from other sources (including the web). Please note that it is compulsory to follow figure instructions. The expression of skin sensitivity can exhibit distinct variations across different anatomical regions, with the facial area frequently exhibiting a heightened susceptibility attributed to its distinctive attributes and continuous exposure to environmental influences. While sensitivity may manifest in other bodily locales, including the hands, scalp, feet, neck, torso, and back, the facial region necessitates a more specialized and attentive approach due to the relatively thinner nature of facial skin in comparison to other anatomical sites (2, 12). The facial region is characterized by an increased density of nerve endings, rendering it highly responsive to stimuli, and consequently more susceptible to sensations of discomfort, itching, and irritation. Furthermore, the face is subject to continuous exposure to various environmental elements, including ultra-violet (UV) radiation, pollution, wind, fluctuations in temperature, and the use of cosmetics and skincare products. This perpetual exposure heightens the vulnerability to sensitivity reactions, as these external factors can disrupt the skin’s protective barrier function and induce inflammation. Insufficient natural oil production in specific areas can lead to moisture imbalances, further exacerbating skin sensitivity (2, 12, 13).

#### 3.5.1 Expert recommendation

From the expert committee meeting, it was gleaned that approximately 50 to 60% of patients exhibit symptoms of dry sensitive skin, affecting various body regions including the hands, feet, scalp, neck, torso, back, and genital area. The committee also noted a gender-specific prevalence, with sensitive skin occurring predominantly in females. In contrast, dry skin is more commonly observed in the geriatric population, indicating a demographic variation in skin sensitivity and condition.

### 3.6 Understanding the spectrum of sensitive skin within the Indian population

Sensitive skin is influenced by many triggering factors that can provoke heightened reactions and discomfort. The Indian climate, characterized by its diverse geography and varying climates across regions, also contributes to the complex nature of sensitive skin. The use of various cosmetics and perfumes, often containing potent fragrances and potentially irritating ingredients, can disrupt the skin’s delicate balance and trigger adverse responses. Cultural influences play a substantial role in the perception and management of sensitive skin in India. This cultural perspective affects the choice of skincare products and treatments for individuals with sensitive skin. The cultural preference for fair skin in India has led to a widespread desire for lighter skin tones. This preference has, unfortunately, driven many individuals to use a variety of cosmetic and skin-lightening products that can potentially sensitize the skin. These products often contain ingredients such as hydroquinone, steroids, and other harsh chemicals that can disrupt the skin’s natural barrier function and lead to adverse reactions. Additionally, stress, a prevalent aspect of modern living, dietary choices, smoking, and tight-fitting fabrics used as garments have been linked to exacerbating skin sensitivity (1, 2).

#### 3.6.1 Pathophysiology of sensitive skin

Despite the presence of typical symptoms indicating inflammation and the involvement of peripheral innervation, the underlying pathophysiology of sensitive skin remains elusive. Buhe et al. conducted a study that involved a comparison of the skin structure between individuals with sensitive skin and those without sensitivity. The findings revealed that the group with sensitive skin displayed a decreased concentration of intraepidermal nerve fibers, specifically peptidergic C-fibers. These fibers play a role in the perception of pain, itching, and temperature, and their deterioration may contribute to the development of allodynia and related symptoms. The findings of this study indicate that the underlying mechanisms of skin sensitivity bear resemblance to those of neuropathic pruritus in the context of small-fiber neuropathy. Furthermore, it is suggested that environmental factors can influence the innervation of the skin (14).

##### 3.6.1.1 Role of neurogenic inflammation and genes in skin sensitivity

The nervous system’s role in sensitive skin is pivotal. Nerves in the skin, known as cutaneous sensory nerves, are responsible for transmitting signals related to touch, temperature, and pain. Individuals with sensitive skin tend to have an enhanced response to stimuli. Within the epidermis, dynamic interplay occurs between nerve fibers and keratinocytes, with significant implications for skin health (4, 15, 16). Genetic factors play a significant role in determining an individual’s susceptibility to sensitive skin. Certain genetic variations can influence skin barrier function, immune response, and sensory perception. Genetic variations can influence the composition of the skin’s outermost layer, the SC. A compromised barrier can lead to increased sensitivity and susceptibility to irritation. Mutations in genes responsible for maintaining the skin’s lipid barrier, such as filaggrin (FLG), can lead to a compromised skin barrier that allows irritants and allergens to penetrate more easily, triggering skin reactions. FLG is a vital protein responsible for the aggregation of keratin filaments, organizing lamellar lipid bilayers, preserving adequate hydration, and balancing the pH of the epidermis. The FLG gene is encoded by chromosome number 1q21. FLG mutations are less commonly found in Indian patients (35–40%) than in the Western population (60–70%). The prevalence of FLG mutation in hand eczema cases was 33.7% of cases in the Indian population (17, 18). In a prospective case–control study of 90 children aged 1–12 years, all diagnosed with various allergic diseases, a significant correlation emerged between FLG mutations and an increased risk of asthma. This correlation was consistent with findings from both the Avon Longitudinal Study of Parents and Children (ALSPAC) and the International Study on Allergy and Asthma in Childhood (ISAAC). Notably, the study identified a 5% prevalence rate of the R501X mutation within the FLG gene among the participating children (19).

### 3.7 Clinical characteristics and diagnostic criteria for sensitive skin

Sensitive skin can manifest as cutaneous symptoms of different skin disorders, such as rosacea, atopic dermatitis, psoriasis, allergic contact dermatitis, and seborrheic dermatitis. Other conditions associated with dry sensitive skin include keratosis pilaris, perioral dermatitis, photosensitive dermatosis, periorbital dermatitis, drug-induced dryness, and skin dryness due to the presence of underlying systemic diseases, such as diabetes and hypothyroidism (20).

#### 3.7.1 Barrier failure in atopic dermatitis

Atopic dermatitis, commonly known as eczema, is a chronic inflammatory skin condition that affects millions of people worldwide. One of the hallmark features of atopic dermatitis is compromised skin barrier function. The skin barrier in atopic dermatitis is characterized by a deficiency in key components that ensure its integrity. Specifically, there is a reduced production of ceramides, fatty acids, and cholesterol (21). Rosacea is a chronic skin condition that manifests facial redness, visible blood vessels, swelling, and in some cases, the formation of pustules and papules. While the exact cause of rosacea remains elusive, recent research has highlighted the role of the skin barrier in its pathophysiology. One of the notable features of the skin barrier in rosacea is increased permeability. This heightened permeability can trigger inflammatory responses and exacerbate the symptoms associated with rosacea, leading to redness, swelling, discomfort, increased sensitivity, dryness, and susceptibility to irritants. Another contributing factor to the disruption of the skin barrier is Demodex infestation (22).

#### 3.7.2 Diagnosis of sensitive skin

Sensitive and reactive skin are indeed closely related but differ significantly in their severity and clinical presentation (23, 24). Sensitive skin primarily manifests as discomfort or unpleasant sensations, such as stinging or burning, without visible signs of inflammation. In contrast, reactive skin involves both sensory symptoms and observable inflammatory responses, such as redness, scaling, and swelling. This condition can arise from environmental factors or the prolonged use of irritating skincare products, leading to more pronounced clinical manifestations. The phenomenon of sensitive skin is predominantly diagnosed through the subjective perceptions of the patients, as it lacks concrete and measurable clinical signs. Unlike many medical conditions that exhibit clear and documentable physical manifestations, sensitive skin often lacks visible indicators. This characteristic poses a challenge for dermatologists seeking to assess and address sensitive skin conditions. Consequently, self-assessment questionnaires, as presented in Table 2, emerge as valuable and validated tools for effectively identifying individuals with sensitive skin (4, 5, 23).

**Table 2:** Questionnaire for diagnosing sensitive skin.

Question	Response
How do you describe your skin?	Oily/dry/combination
Is your facial skin easily irritated?	Yes/no
Apart from the face, is there any other site that is reddened/easily irritated?	Yes/no
Have you experienced an adverse reaction to a cosmetic product?	Never/sometimes/often/always
Do some cosmetic products make your skin burn or irritate?	Never/sometimes/often/always
Do some cosmetic products make your skin itch within 30 min?	Never/sometimes/often/always
Do some cosmetic products make your skin sting within 30 min?	Never/sometimes/often/always
Have you experienced an adverse reaction to a cosmetic product in the last 12 months?	Never/sometimes/often/always
Have you ever experienced eczema or dermatitis?	Yes/no
Do you have eczema or dermatitis at the moment?	Yes/no
Do some hair products make your scalp itch, sting, or become red?	Never/sometimes/often/always
Did you experience eczema or dermatitis during your childhood?	Yes/no
Have you ever experienced asthma or hay fever?	Yes/no
Do you tend to experience dandruff?	Never/sometimes/often/always
Does your skin get easily reddened and irritated by environmental factors? (a) Variations in temperature; (b) Pollution; (c) Air conditioning; (d) Water	Never/sometimes/often/always

The experts recommended validation of the questionnaire through patient participation. While self-assessment questionnaires indeed remain a reliable method for diagnosing sensitive skin, several physical tests can provide valuable insights that aid specialists in arriving at a precise and accurate diagnosis. These tests contribute to a comprehensive diagnostic approach by offering objective data that complements the patient’s subjective experiences, enhancing the overall diagnostic accuracy and confidence (4, 5, 25). The various tests used to diagnose sensitive skin are enumerated in Tables 3, 4 (5, 25).

**Table 3:** Diagnostic tests for identification of sensitive skin.

In-office test	Procedure	Interpretation	Advantages	Disadvantages
Lactic acid sting test	10% lactic acid (0.5 mL) is applied to the nasolabial groove.	A stinging sensation was reported by the patient within minutes of the application.	Fast and economical procedure	Poor reproducibility
Chloroform methanol test	Application of 20:80 solution of chloroform: methanol at malar prominence.	A burning sensation was reported by the patient.	Quick procedure	Poor reproducibility
Sensory perception threshold management test	Application of capsaicin (0.075%)	Altered unmyelinated C fibers result in individuals with sensitive skin having a decreased perception threshold.	Fast procedure	Limited consistency
Irritant reactivity test	Application of sodium lauryl sulfate at the forearm	Skin irritation after exposure to sodium lauryl sulfate	Easy and reasonable procedure	Limited consistency

**Table 4:** Bio-engineering tests.

Test	Parameters analyzed	Method
Evaporimetry (TEWL analysis)	Analyzes skin barrier dysfunction (water evaporation through the barrier)	Closed, open, ventilated chamber method.
Corneometry	Measures skin hydration	A capacitance measuring device operating at low frequency (40–75 Hz) is used to measure water content in the epidermis.
Profilometry	Analyses skin surface texture	Silicon rubber cast of the skin surface is obtained, the cast is transformed into plastic positive, and computerized tracing is obtained.
Squametry	Analyses skin surface texture	Sticky tape is placed against loosely adherent skin and tape is analyzed through computerized image processing.
A-scan ultrasound	Analyses of changes in skin thickness	A-scan ultrasound.

TEWL: Transepidermal water loss.

The investigation for sensitive skin diagnosis involves a combination of patient history, trigger identification, physical examination, self-assessment questionnaires, and potentially patch testing. This multifaceted approach enables dermatologists to comprehensively evaluate individuals experiencing skin sensitivity, leading to accurate diagnosis and personalized management strategies (5, 25). An expert recommendation on clinical diagnostic criteria for sensitive skin is outlined in Table 5.

**Table 5:** Expert recommendations on clinical diagnostic criteria for sensitive skin.

**Expert recommendations on clinical diagnostic criteria for sensitive skin**
Most panelists diagnosed sensitive skin primarily through the observation of a constellation of signs and symptoms, which included redness, dryness, scaling, peeling, the appearance of bumps and hives, along with sensations of burning, tightness, itching, stinging, pain, and dysesthesia. The expert group strongly recommends the utilization of a standardized questionnaire to assess self-reported cases of sensitive skin, avoiding direct inquiries to prevent reporting bias and ensure accurate, unbiased symptom assessment. Integrating objective tests, specifically the SLS challenge, TEWL measurement using a Tewameter®*, and the lactic acid sting test, is recommended to enhance the assessment quality for sensitive skin. These tests provide objective, quantifiable data that complements self-reported symptoms. The Pons Guiraud and Muizzuddin classification systems can be incorporated into clinical practice for a comprehensive assessment of sensitive skin, aligning with the physician’s evaluation.

SLS: Sodium lauryl sulfate; TEWL: Transepidermal water loss. *A scientific instrument used to measure TEWL.

**Figure 2:**
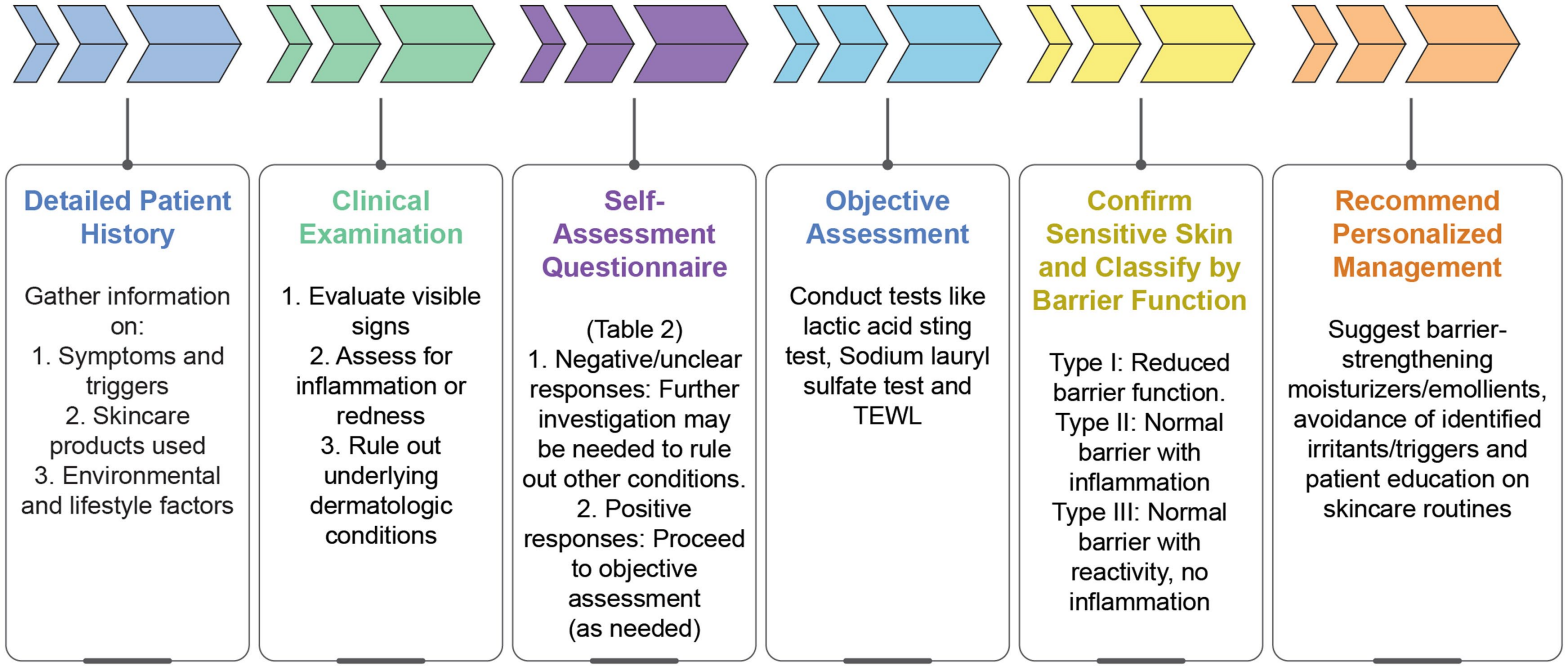
Diagnostic algorithm for sensitive skin. TEWL: Transepidermal water loss.

Figure 2 presents a practical diagnostic algorithm based on expert recommendations and available scientific evidence, facilitating structured clinical assessment and diagnosis of sensitive skin.

### 3.8 Management of sensitive skin

**Figure 3:**
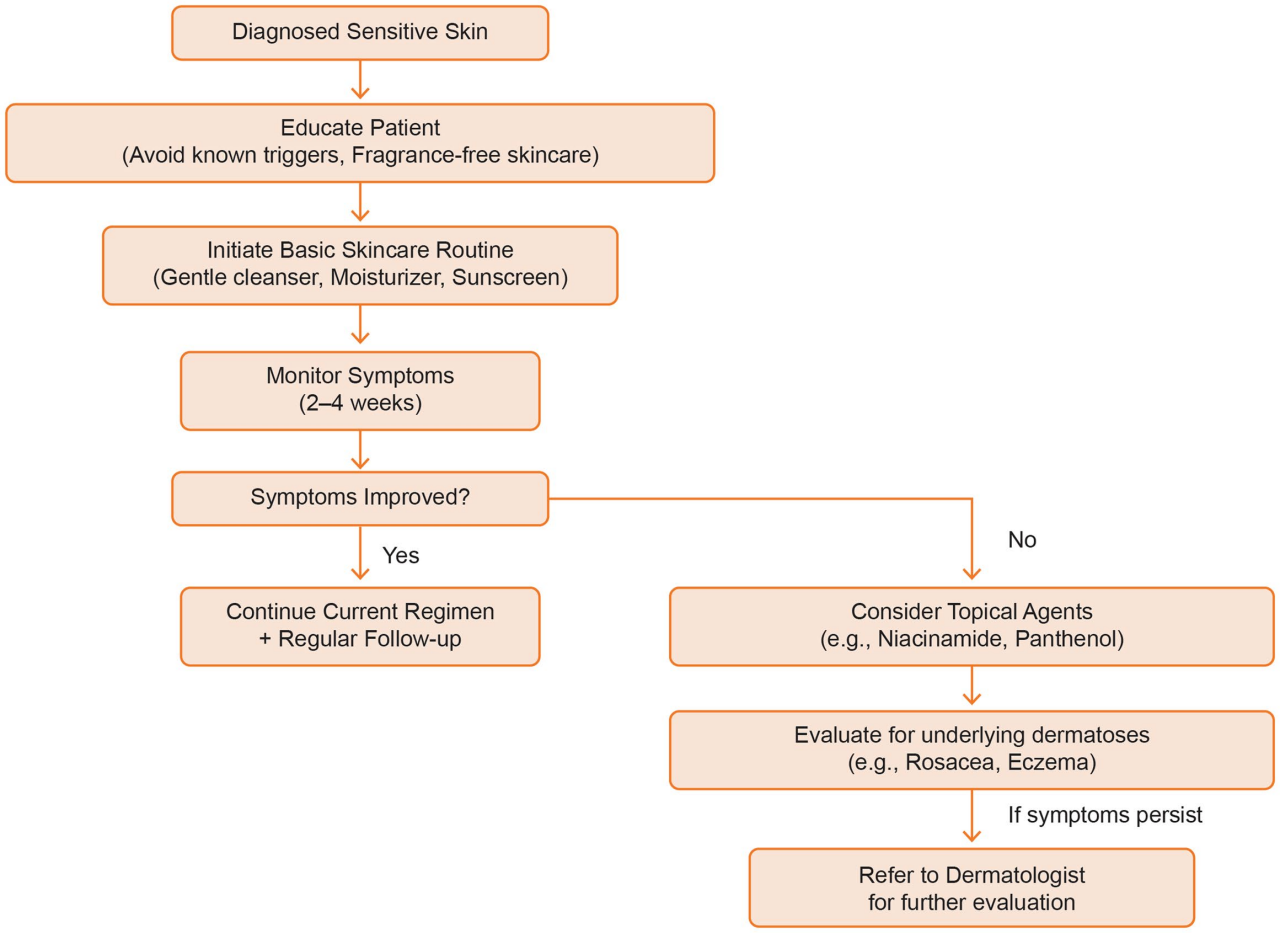
Therapeutic algorithm for sensitive skin.

Currently, there are no specific protocols designed to manage sensitive skin. The management of sensitive skin needs proper guidelines to preserve the skin’s homeostasis, mitigate reactivity, and bolster overall dermal health. Key considerations encompass the reinforcement of the SC barrier, amelioration of irritation responses, and the promotion of optimal epidermal function (5). Figure 3 outlines a therapeutic algorithm based on expert recommendations and clinical experience, offering a structured approach to the personalized management of sensitive skin according to symptom severity and treatment response.

#### 3.8.1 Approach for formulating and selecting the right cosmetic product for sensitive skin

The formulation of sensitive skin products is indeed a challenge as there is a variety of marketed cosmetic products and toiletries that claim to be “safe for sensitive skin,” “hypoallergenic,” “clinically tested,” “dermatologist-tested,” “completely natural,” “unscented,” and “fragrance-free.” Addressing the unique needs of dry and sensitive skin in the Indian context requires targeted cosmetic formulations that provide effective moisturization and soothing properties without causing irritation. India’s diverse climate and environmental factors can exacerbate skin dryness and sensitivity, necessitating the formulation of products that cater to these challenges. Draelos et al. demonstrated that the formulation of sensitive skin products should involve reviewing the composition of the product to rule out the presence of any potent irritant or sensitizer, eliminating unnecessary components from the product for better compatibility, in-depth evaluation with *in vitro* methods for predicting the product’s potential for skin and eye irritation, and *in vivo* methods to validate skin irritation and sensitizations (25). The desired properties of skin cleansers for acne, atopic dermatitis, and rosacea are that they should be non-comedogenic, non-irritating, have a mildly acidic pH, be fragrance-free, and should have properties to improve the skin barrier. Similarly, moisturizers for different skin diseases associated with sensitive skin syndrome should restore the skin barrier, have skin hydration potential, are fragrance-free, non-allergic, and non-acnegenic. Effective photoprotection plays a pivotal role in safeguarding the skin against sensitivity, sunburn, and premature aging caused by sun exposure, and the exacerbation of underlying skin conditions. This is particularly critical in Asian populations, predominantly those with Fitzpatrick skin types IV and V, as they have a heightened susceptibility to post-inflammatory hyperpigmentation, making sun protection even more essential. A broad-spectrum sunscreen with a sun protection factor (>30) should be recommended by the dermatologist after understanding different skin types in the Indian population as different skin types react differently to various topical skin products. Cosmetic formulations for Indian dry sensitive skin should undergo rigorous testing to ensure their hypo-allergenicity and efficacy (5, 26).

##### 3.8.1.1 Cosmeceuticals

In the world of skincare and beauty, a novel and dynamic term has emerged to define products that straddle the line between cosmetics and pharmaceuticals: cosmeceuticals. Cosmeceuticals incorporate biologically active ingredients, blurring the distinction between traditional cosmetics and pharmaceuticals, and providing an additional approach to dermatological management. They are formulated to deliver dermatological benefits beyond basic skincare, and to provide measurable skin benefits at a cellular level. Cosmeceuticals encompass a wide range of products, including creams, serums, lotions, and masks, which are formulated to address specific skin concerns. Unlike conventional cosmetics, cosmeceuticals often contain bioactive compounds, such as antioxidants, peptides, vitamins, and growth factors. They encompass a wide range of therapeutic and preventive applications for the management of skin disorders such as atopic dermatitis, rosacea, and psoriasis. With India’s diverse population and specific skin concerns, such as hyperpigmentation, melasma, and acne, these products offer targeted therapeutic options. For a range of skin conditions. In conditions such as acne, rosacea, and hyperpigmentation, cosmeceuticals containing active ingredients, such as salicylic acid, niacinamide, and alpha hydroxy acids, have demonstrated efficacy in reducing inflammation, controlling sebum production, and promoting skin cell turnover (27, 28). Based on dermatologists’ opinions, a few cosmeceutical products can be included in the daily skin care regimen, such as moisturizers, toners, cleansers, sunscreens, and serums (29).

##### 3.8.1.2 Colloidal oatmeal–based moisturizers

Colloidal oatmeal–based moisturizers have emerged as a beneficial therapeutic option for managing sensitive skin in India. Colloidal oatmeal, derived from finely ground oats (*Avena sativa*), has gained recognition for its potential therapeutic properties, particularly in skincare and dermatology. Colloidal oatmeal consists of sugar, amino acids, protein, lipids, and fiber. These bioactive constituents intricately contribute to the reinforcement of the stratum corneum, the reduction of trans-epidermal water loss, the manifestation of potent anti-inflammatory effects, and the attenuation of susceptibility to cutaneous irritation. The United States Food and Drug Administration (USFDA) approved oatmeal as a safe and effective over-the–counter (OTC) drug in 1989 (30). Colloidal oatmeal has several benefits beyond moisturization, such as anti-inflammatory, anti-pruritic, anti-mitotic, and pre-biotic properties. They repair the skin barrier by restoring the pH of the skin (29–31). Colloidal oatmeal-based emollients are formulated with finely ground oat particles suspended in a liquid medium. These emollients are gaining recognition for their combination of skin–soothing properties and moisturizing benefits. Additionally, colloidal oatmeal also hydrates the skin and maintains the pH of the skin surface. Furthermore, colloidal oatmeal can effectively disrupt the itch–scratch cycle, a detrimental pattern in which scratching-irritated skin intensifies itching, leading to more scratching. This intervention is accomplished by establishing a protective barrier on the skin, which not only eases itching but also fosters skin healing, thereby enhancing the quality of life for patients with skin disorders (31–33).

The use of corticosteroids (which is an active ingredient in many cosmeceutical moisturizers) is associated with an increased risk of complications involving stria, hypertrichosis, telangiectasia, and skin atrophy. To minimize the adverse events associated with the use of corticosteroids, colloidal oatmeal-based emollients are used as a single-agent therapy to manage dry sensitive skin in children and young adults with atopic dermatitis. Colloidal oatmeal is a cost-effective treatment for different skin conditions, especially if used early in the treatment. Colloidal oatmeal can help to reduce healthcare utilization costs and reduce the need for steroid and antibiotic usage (31, 32, 34). Between April 2019 and November 2020, the Short-term Topical Application to Prevent Atopic Dermatitis (STOP AD) randomized controlled trial was conducted at Cork University Maternity Hospital (CUMH), recruiting high-risk newborn infants for atopic dermatitis prevention. The study showed a significant reduction in atopic dermatitis by daily application of the colloidal oat-based emollient (33). Another study involving 54,000 patients showed that the use of an emollient containing colloidal oatmeal led to a 39.4% reduction in the need for corticosteroids and a 21% decrease in the use of antibiotics. Colloidal oatmeal-based emollients offer a holistic approach to skincare, combining moisturizing and protective benefits. With their ability to address a spectrum of skin concerns, such as atopic dermatitis, psoriasis, and acne, while being well-tolerated by sensitive skin types, these emollients offer an effective option for promoting skin health and maintaining a natural skin barrier. Emollients containing components such as humectants, skin conditioning agents, and ceramides function to hydrate and restore the integrity of a damaged and dry skin barrier. Colloidal oatmeal-based moisturizers have been proven to be valuable for combating dry, sensitive skin conditions because the oat lipid extract can activate peroxisome proliferator-activated receptors (PPAR). They are regulators known to enhance epidermal barrier function. Additionally, treatment with oat oil led to a substantial increase in the expression of ceramide-processing genes. Notably, oat oil treatment resulted in a remarkable 70% increase in ceramide levels, indicating the functional impact of PPAR activation by oat oil on keratinocytes and, consequently, an improvement in skin barrier function (31, 32, 35).

##### 3.8.1.3 Non-oatmeal–based moisturizers

Moisturizers that are not oatmeal-based encompass a wide array of skincare products designed to hydrate and nourish the skin without utilizing oat-derived components. These formulations leverage various ingredients and mechanisms to deliver effective moisturization and address specific skin concerns. Non-oatmeal–based moisturizers may not provide the same extent of barrier repair as oatmeal-based products as they may contain ingredients that can irritate sensitive skin types. These ingredients can include fragrances, preservatives, or harsh chemicals. Furthermore, non-oatmeal–based moisturizers have certain drawbacks and limitations, including potential irritants, high cost, varying formulations, and the absence of specific skin-soothing properties found in oatmeal-based emollients. Experts recommended that non-oatmeal–based moisturizers can be applied in liberal amounts as they are inexpensive and hence suitable for patients from low socioeconomic backgrounds, resulting in adherence to treatment. Different non-oatmeal–based moisturizers and their mechanisms are detailed in Table 6 (31, 36, 37).

**Table 6:** Non-oatmeal–based moisturizers.

Non-oatmeal–based moisturizers	Mechanism
Glycerin-based emollient	Non-greasy formulas that hydrate the skin.
Glycyrrhetinic acid-containing barrier cream	Anti-inflammatory and soothing properties help to protect the natural skin barrier.
Ceramide–based emollient	Maintains a healthy skin barrier, improves moisture retention, and reduces skin sensitivity.
Hyaluronic acid-based emollient	Deeply hydrates the skin and makes it supple.
Mineral oil, petrolatum, and paraffin-based moisturizer	Hydrates the skin and prevents skin dryness.

The experts’ recommendations on cosmeceuticals are outlined in Table 7.

**Table 7:** Expert recommendation on cosmeceuticals.

**Expert Recommendation on Cosmeceuticals**
Management approaches for sensitive skin should focus on a cleansing and moisturization regimen. Additionally, the continual use of complete emollient therapy involving a combination of emollients and emollient-based soap substitutes is effective in the management of sensitive skin. The panel emphasized that the inclusion of ceramides in moisturizers enhances their therapeutic efficacy. It was specifically noted that a moisturizer formulated with both ceramide and colloidal oatmeal, which aids in increasing endogenous ceramide production, is preferable for achieving a more sustained and effective result in skin care. Moisturizers, including those with colloidal oatmeal or ceramides, can be prescribed for various skin concerns. The choice should be guided by factors such as the severity of skin dryness, the extent of lesions, the stage of the condition, the patient’s socioeconomic circumstances, and prevailing climatic conditions. Colloidal oatmeal offers ceramide-boosting, barrier-strengthening, prebiotic, and soothing properties, making it a valuable option in certain scenarios.

### 3.9 Approach for the management of sensitive skin in diverse patient profiles

The “2 weeks” strategic guidelines in practice for managing individuals with different skin conditions along with skin sensitivity involve discontinuing all OTC cosmetics, cleansers, and other products (topical medications, especially those containing tretinoin, volatile alcohols, glycolic acid, or other skin irritants) collectively referred to as “skin diet” for 2 weeks. After an initial two-week period, it is essential to assess the patient for the potential presence of underlying skin disorders, including seborrheic dermatitis, acne, and psoriasis. Should any clinical indicators persist beyond this assessment, the appropriate course of action entails initiating a two-week treatment regimen with suitable medications. Subsequently, to exclude the possibility of allergic contact dermatitis or contact urticaria, it is recommended to conduct patch or photo patch tests, followed by a facial string test. The dermatologist should comprehensively assess all findings, regardless of their nature (whether positive or negative), and subsequently tailor the selection of ingredients or products for each patient, determining which should be either continued or discontinued (5, 25). The approach for the management of patients with diverse skin conditions is presented in Supplementary Table S1 (38–48).

#### 3.9.1 Additional approaches for the treatment of sensitive skin

Research has recently discovered that trans-4-tert–butylcyclohexanol, a selective antagonist, effectively inhibits the activation of human transient receptor potential vanilloid type 1 (hTRPV1) by capsaicin, a natural TRPV1 activator. This was demonstrated in experiments using human embryonic kidney (HEK293) cells with hTRPV1. In a clinical trial involving 30 women using capsaicin-containing emulsions, applying a topical treatment containing 0.4% of this antagonist significantly reduced capsaicin-induced burning sensation on the skin (*p* < 0.0001). These findings suggest that trans-4-tert–butyl cyclohexanol holds promise as a potential bioactive for managing sensitive skin. In another study, researchers utilized a probiotic lysate derived from *Bifidobacterium longum* extract to address sensitive skin. They first demonstrated its efficacy *in vitro* and then confirmed its benefits in a clinical trial. A 10% topical cream containing this extract was tested on 66 female volunteers in a randomized, double-blind, placebo-controlled trial. Results from clinical and self-assessments showed a significant reduction in skin dryness after 29 days among participants using the cream with the active extract. Although both studies have shown promising results, further research is warranted to determine the best molecule that can address the problems of sensitive skin (5, 49). Table 8 elucidates the expert’s recommendations on the management of patients with sensitive skin.

**Table 8:** Expert recommendations on the management of patients with sensitive skin.

**Expert recommendations on the management of patients with sensitive skin**
Emollients play a key role in the management of various skin conditions such as dry skin, itchy skin, acne, rosacea, atopic dermatitis, steroid-induced skin damage, conditions related to oncology treatments, ichthyosis, seborrheic dermatitis, hand eczema, psoriasis, diabetes-related dryness, keratosis pilaris, perioral dermatitis, photosensitive dermatoses, and periorbital dermatitis. The moisturizers should be affordable and convenient to use with easy spreadability. For extensive body lesions, non-oat-based moisturizers may be preferred due to their cost-effectiveness, which enhances patient compliance. However, for non-extensive and facial-sensitive skin, ceramide or oat-based emollients may be prescribed. Antihistamines may be prescribed in the management of sensitive skin involving pruritus. All emollients including ones with Ceramides or oatmeal can be considered for managing sensitive skin conditions.

##### 3.9.1.1 Key findings from the expert’s recommendation

- Emollients should be chosen based on the cause of dryness, the phase of the condition (active or maintenance), lesion severity, patient socioeconomic status, and climate. For dry or sensitive skin, emollients with ceramides restore the skin barrier and offer lasting benefits. Colloidal oatmeal-based moisturizers, while not essential, may provide additional benefits for dry and sensitive skin by supporting endogenous ceramide production and strengthening the skin barrier.
- The panel members deliberated on the ambiguity in the published literature, particularly regarding the inconsistent use of terminology in defining sensitive skin. Consequently, they highlighted the necessity for future research, given the scarcity of India-specific data in this context.
- Further research is essential to establish a universally applicable questionnaire for the diagnosis of generalized skin sensitivity in various settings.

## 4 Conclusion

In conclusion, addressing the needs of sensitive skin in India requires a comprehensive understanding of the country’s diverse climate, cultural practices, and prevalent skin conditions. This expert recommendation paper provides a summary of the overview of the evolving landscape of sensitive skin in India, with a particular focus on the substantial gap in the current knowledge base about this condition. This gap predominantly relates to the insufficient availability of India-specific data, as highlighted by expert insights and opinions. The unique combination of factors such as high humidity, extreme temperatures, pollution, and traditional skincare rituals can significantly impact the management of sensitive skin. To cater to this specific demographic, skin care products must be formulated with mild, hypoallergenic ingredients that minimize the risk of irritation and adverse reactions. Experts recommend the use of appropriate cleansers and moisturizers, which are mild, dermatologically tested, and suitable for sensitive skin with barrier-strengthening properties. These products could lead to better patient compliance and hence better patient outcomes, but there is a need to generate data specific to the Indian population. Colloidal oatmeal demonstrates potential benefits in the management of sensitive skin in the Indian context. Its potential to relieve irritation and inflammation, combined with its natural composition, differentiates it from non-colloidal oatmeal products. Nevertheless, the journey toward a more nuanced understanding of its benefits requires continued scientific research. This will not only solidify its position as an effective solution but also guide the formulation of optimal skincare strategies tailored to the unique needs of individuals with sensitive skin in India.
