# Supplementary material for: Spectrum of sensitive skin in India: a collaborative expert position statement
Source: Front Med (Lausanne). 2025 Aug 26;12:1625172. doi: 10.3389/fmed.2025.1625172 (PMC12419229; doi:10.3389/fmed.2025.1625172)
Supplement: Supplementary file 1 [file Table_1.docx]

Supplementary Material

# Supplementary Table

Table S1. Approach for managing patients with specific skin conditions

| **Skin condition** | **Age** | **Gender** | **Signs and symptoms** | **Family history** | **Comorbidities** | **Diagnosis** | **Treatment** |
| --- | --- | --- | --- | --- | --- | --- | --- |
| Atopic dermatitis (38,39) | More prevalent in early childhood followed by the adult group. | More common in males | Dry and itchy skin | Family history of AD or allergy | Hypercholesterolemia, hypertriglyceridemia, diabetes, obesity, rhinitis, asthma | Clinical assessment and  William's criteria | Emollients, topical steroids, and phototherapy |
| Acne (40,41) | More prevalent in teenagers or early childhood followed by the adult group. | More common in females | Small to large papules, pustules, and nodules | History of acne | Cushing syndrome, polycystic ovary syndrome, obesity, and diabetes | Clinical examination | Topical and oral antibiotics, topical steroids, isotretinoin, and hormone therapy |
| Rosacea (42) | 30 to 50 years of age | More common in females | Flushing, erythema, papules, telangiectasia, dry skin | Higher incidence in patients with a family history of rosacea | Neurological disease, inflammatory bowel disease, and cardiovascular diseases | Clinical examination (ophthalmic assessment is mandatory) | Topical antibiotics, systemic antibiotics, artificial tears (ocular involvement), and electrosurgery |
| Psoriasis (43) | 30 to 39 years of age | More common in females | Salmon pink plaques, Auspitz sign, and erythematous rash | More common in patients with a family history of psoriasis | Psoriatic arthropathy and psychological, hepatic, and cardiovascular diseases | 1. PEST  2. PASI  3. Physician Global Assessment Scale | First-line therapy  Vitamin D analogs and topical steroid therapy  Second-line therapy  Phototherapy and PUVA |
| Keratosis pilaris (44) | Common in adults | No specific gender predilection | Small inflammatory papules with follicular involvement and asymptomatic erythematous bumps on the skin (mostly seen in lower extremities) | More common in patients with a family history of keratosis pilaris, AD, and ichthyosis | Down syndrome, Noonan syndrome, obesity,  diabetes, ichthyosis vulgaris, and malnutrition | History and clinical examination | Asymptomatic, usually improves over time. |
| Cosmetics overuse (45) | Above 18 years | More common in females | Pruritis, burning, pungency, tingling, skin thickening, and dryness | Family history of allergy and contact dermatitis | Contact and atopic dermatitis | Clinical examination, self-assessment questionnaire, and contact test | Discontinue all cosmetics for two weeks and prescribe moisturizers and photoprotectors. |
| Dry skin (due to underlying systemic diseases like diabetes) (45, 46) | 20 to 79 years | More prevalent in males | Cutaneous infection, xerosis, and callus | Family history of skin diseases and diabetes | Psoriasis, diabetic dermopathy, and obesity | Clinical examination, fasting blood sugar test, and HbA1C | Managing elevated sugar levels. Applying moisturizers, utilizing emollients containing glycerol, and employing a 10% urea cream. |
| Dry sensitive skin due to oncology treatment (47, 48) | 25 to 70 years | More prevalent in females | Rashes, itching, erythema, and dry desquamation epidermal necrosis | Family history of skin diseases and skin cancer | Radiation dermatitis | Clinical assessment and Skindex-16 questionnaire | Emollients, topical antibiotics, skin cleansers, and photoprotection |

AD: Atopic dermatitis; HbA1c: Glycated haemoglobin; PASI: Psoriasis Area Sensitive Index; PEST: Psoriasis Epidemiology Screening Tool; PUVA: Psoralen plus ultraviolet A radiation.
